# Local extreme map guided multi-modal brain image fusion

**DOI:** 10.3389/fnins.2022.1055451

**Published:** 2022-10-28

**Authors:** Yu Zhang, Wenhao Xiang, Shunli Zhang, Jianjun Shen, Ran Wei, Xiangzhi Bai, Li Zhang, Qing Zhang

**Affiliations:** ^1^School of Astronautics, Beihang University, Beijing, China; ^2^Department of Electronic Engineering, Tsinghua University, Beijing, China; ^3^School of Software Engineering, Beijing Jiaotong University, Beijing, China; ^4^Department of Radiation Oncology, National Cancer Center, National Clinical Research Center for Cancer, Cancer Hospital, Chinese Academy of Medical Sciences and Peking Union Medical College, Beijing, China; ^5^Department of Orthopaedic Oncology, Beijing Jishuitan Hospital, Beijing, China

**Keywords:** multi-modal brain images, image fusion, image guided filter, local extreme map, bright and dark feature map

## Abstract

Multi-modal brain image fusion targets on integrating the salient and complementary features of different modalities of brain images into a comprehensive image. The well-fused brain image will make it convenient for doctors to precisely examine the brain diseases and can be input to intelligent systems to automatically detect the possible diseases. In order to achieve the above purpose, we have proposed a local extreme map guided multi-modal brain image fusion method. First, each source image is iteratively smoothed by the local extreme map guided image filter. Specifically, in each iteration, the guidance image is alternatively set to the local minimum map of the input image and local maximum map of previously filtered image. With the iteratively smoothed images, multiple scales of bright and dark feature maps of each source image can be gradually extracted from the difference image of every two continuously smoothed images. Then, the multiple scales of bright feature maps and base images (i.e., final-scale smoothed images) of the source images are fused by the elementwise-maximum fusion rule, respectively, and the multiple scales of dark feature maps of the source images are fused by the elementwise-minimum fusion rule. Finally, the fused bright feature map, dark feature map, and base image are integrated together to generate a single informative brain image. Extensive experiments verify that the proposed method outperforms eight state-of-the-art (SOTA) image fusion methods from both qualitative and quantitative aspects and demonstrates great application potential to clinical scenarios.

## 1. Introduction

With the development of the medical imaging techniques, patients are often required to take multiple modalities of images, such as computed tomography (CT), magnetic resonance (MR) image, positron emission tomography (PET), and single-photon emission computed tomography (SPECT). Specifically, CT image mainly captures dense structures, such as bones and implants. MR image can capture soft-tissue information clearly, such as muscle and tumor. PET image can help reveal the metabolic or biochemical function of tissues and organs and SPECT image can visualize the conditions of organs, tissues, and bones through delivering a gamma-emitting radioisotope into the patient. Then, through observing all these captured medical images, the doctors can precisely diagnose the possible diseases. However, accurately locating the lesions and diagnosing the corresponding diseases from multiple modalities of images are still complex and time-consuming for the doctors. Therefore, the image fusion technique can be applied to merge the salient and complementary information of the multi-modal images into a single image for better perception of both doctors and intelligent systems (Yin et al., 2018; Liu et al., 2019, 2022a,b,c,d; Xu and Ma, 2021; Wang et al., 2022).

In recent years, many methods have been proposed for the task of multi-modal image fusion. Generally, these methods can be divided in two categories, i.e., spatial-domain image fusion methods and transform-domain methods (Liu et al., 2015, 2018; Zhu et al., 2018, 2019; Yin et al., 2019; Xu et al., 2020a; Zhang et al., 2020). Specifically, the spatial-domain image fusion methods first decompose the source images into multiple regions, and then integrate the salient regions together to generate their fusion image (Bai et al., 2015; Liu et al., 2017, 2018, 2020; Zhang et al., 2017). The fusion images of these methods often yield unsatisfactory effect due to their inaccurate segmentation results. The transform-domain methods are more popular in the field of image fusion. These methods first convert the source images into a specific domain, then fuse the salient features in this domain, and finally generates the fusion image by converting the fused features back to the image domain (Liu et al., 2015; Xu et al., 2020a; Zhang et al., 2020). The fusion images of these methods are usually more suitable for human to perceive, but might suffer from the blurring effect (Ma et al., 2019a). Moreover, with the fast development of deep-learning techniques, many deep-learning (mainly convolutional neural network, CNN) based image fusion methods have been proposed (Liu et al., 2017; Li and Wu, 2018; Ma et al., 2019b; Wang et al., 2020; Zhang et al., 2020). These methods adopt CNN to extract the deep convolutional features, then fuse the features of the source images by a feature fusion module, and finally reconstruct the fused features as their fusion images. Even though these deep-learning based methods have achieved great success in the field of image fusion, many of these methods would generate fusion images of low contrast or having other kinds of defects.

Amongst the transform-domain methods, the guided image filter (He et al., 2012) demonstrates to be a state-of-the-art (SOTA) edge-preserving image filter, and has been widely used in the field of image fusion (Li et al., 2013; Gan et al., 2015). But in these methods, the guided image filter is often used to refine the decision map or weight map rather than used to extract salient features due to its relatively weak ability in feature extraction. Therefore, in this study, we aim to improve the feature extraction ability of the guided image filter, and based on our improved guided filter to further develop a multi-modal brain image fusion method.

To be specific, we have developed a local extreme map guided image filter, which consists of a local minimum map guided image filter and a local maximum map guided image filter. The developed local extreme map guided image filter is able to more effectively smooth the input image as compared to the original image filter guided by the input image itself, then the features extracted from the difference image of the smoothed image and input image by our filter will be naturally more salient than those extracted by the original image filter guided by the input image itself. Through extending the local extreme map guided filter to multiple scales, we propose a local extreme map guided image filter based multi-modal brain image fusion method. Specifically, we first apply the local extreme map guided image filter iteratively on each source image to extract their multi-scale bright and dark feature maps. Then, the multi-scale bright feature maps, multi-scale dark feature maps, and the base images of the multi-modal brain images are fused, respectively. Finally, the fused bright feature map, dark feature map, and base image are integrated together to generate our fused brain image.

The contributions of this study can be concluded in three parts:

- We propose a new scheme to improve the feature extraction ability of the guided image filter, i.e., using two guided image filters with a local minimum map and a local maximum map, respectively, as their guidance images. This scheme can be incorporated with various guided filters or other similar filters in pursuit of improving their feature extraction ability.
- Based on the local extreme map guided image filter, we further propose an effective image fusion method for fusing multi-modal brain images. Moreover, the proposed method can be easily adapted to fuse other modalities of images while achieving superior fusion performance.
- Extensive experiments verify that our method performs comparably to or even better than eight SOTA image fusion methods (including three conventional methods and five deep learning based methods) in terms of both qualitative and quantitative evaluations.

The rest of this paper is organized as follows. In Section 2, the constructed local extreme map guided image filter and the proposed multi-modal brain image fusion method are elaborated, respectively. Then, the experimental results and discussions are made in Section 3. Finally, this study is concluded in Section 4.

## 2. Proposed method

**Figure 1:**
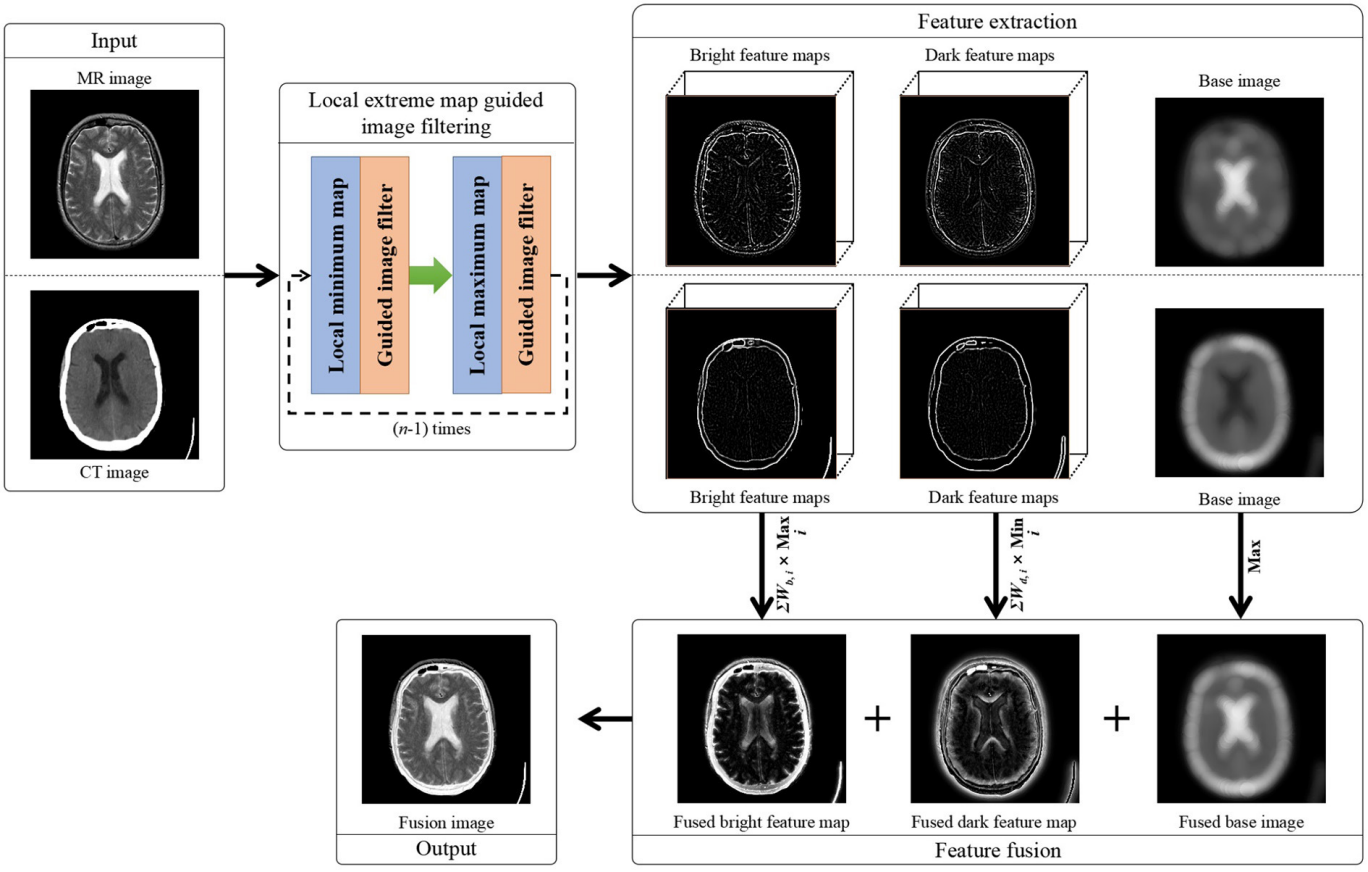
Flowchart of our proposed local extreme map guided multi-modal brain image fusion method. (Note that the dark feature maps in this figure have been illustrated as their absolute feature maps in order to properly visualize the dark features).

The overall structure of the proposed method is illustrated in Figure 1. The major procedures of the proposed method include: First, the two multi-modal brain image are iteratively smoothed by the local extreme map guided filter, respectively. Then, different scales of bright and dark feature maps are extracted, respectively, from the two multi-modal brain images, and the two smoothed brain images in the last iteration are taken as their base images, respectively. Afterwards, each scale of bright feature maps of the two brain images and each scale of dark feature maps of the two brain images are fused by selecting their elementwise maximum values and their elementwise minimum values, respectively. Further, the fused multi-scale bright feature maps and dark feature maps are integrated as a single bright feature map and a single dark feature map, respectively, and the two base image are fused as their elementwise maximum values as well. Finally, the fusion image is generated by integrating the fused bright feature map, dark feature map, and base image together. In the following subsections, the local extreme map guided image filter and our proposed image fusion are elaborated, respectively.

### 2.1. Local extreme map guided image filter

In the guided image filter based image fusion methods (Li et al., 2013; Gan et al., 2015), the guided image filter was often used to adjust the decision maps or weight maps for fusing the feature maps of input images rather than directly extracting the salient feature maps from the input images, due to its limited feature extraction ability. Therefore, in this study, we focus on improving the feature extraction ability of the guided image filter by designing appropriate guidance images.

In the official demonstration of guided image filter (He et al., 2012), the input image is smoothed under the guidance of the input image itself to approach the edge preserving effect. However, in this way, the feature map generated by subtracting the filtered image from the input image is usually not salient enough for the task of image fusion. In order to enhance the feature map, we have modified the guidance image from the input image to its local extreme maps, so that the salient features of the input image can be largely suppressed and accordingly these salient features can be effectively extracted from the difference image of the input image and filtered image. The detailed construction method of our local extreme map guided image filter is described as follows.

First, the input image is filtered under the guidance of the local minimum map of the input image as:


(1) $$\begin{array}{l} I_{f}^{\prime }=guidedfilter\;\left(I,I_{\min},r\right), \end{array}$$

where *guidedfilter* denotes the guided filter (He et al., 2012). *I* and *I*_min_ are the input image and guidance image, respectively. *r* denotes the size of the local window for constructing the linear model between input image and guidance image. Moreover, *I*_min_ denotes the local minimum image of *I*. Under the guidance of the local minimum map, the salient bright features could be sufficiently removed from the input image. Specifically, *I*_min_ can be solved by the morphological erosion operation as:


$$\begin{array}{l} I_{\min}=imerode\;\left(I,se\right), \end{array}$$

where *imerode*(·) denotes the morphological erosion operator. *se* denotes the structuring element of flat-disk shape, radius of which is denoted by *k*.

Then, $I_{f}^{\prime }$ is further filtered under the guidance of its local maximum map as:


(2) $$\begin{array}{l} I_{f}=guidedfilter\;\left(I_{f}^{\prime },I_{\max},r\right), \end{array}$$

where $I_{f}^{\prime }$ and *I*_max_ are the input image and guidance image, respectively, and *I*_max_ denotes the local maximum image of $I_{f}^{\prime }$. Under the guidance of the local maximum map, the salient dark features could be further removed from the finally filtered image. Similar to the solution of *I*_min_, *I*_max_ can be efficiently solved by the morphological dilation operation as:


$$\begin{array}{l} I_{\max}=imdilate\;\left(I_{f}^{\prime },se\right), \end{array}$$

where *imdilate*(·) denotes the morphological dilation operator.

In order to conveniently introduce the following image fusion method, we denote by *leguidedfilter*(·) the function of our constructed local extreme map guided image filter [composed by Equations (1) and (2)], then smoothing an image with the local extreme map guided image filter can be expressed as:


(3) $$\begin{array}{l} I_{f}=leguidedfilter\;\left(I,se,r\right), \end{array}$$

where *r* and *se* correspond to the parameters in Equations (1) and (2).

**Figure 2:**
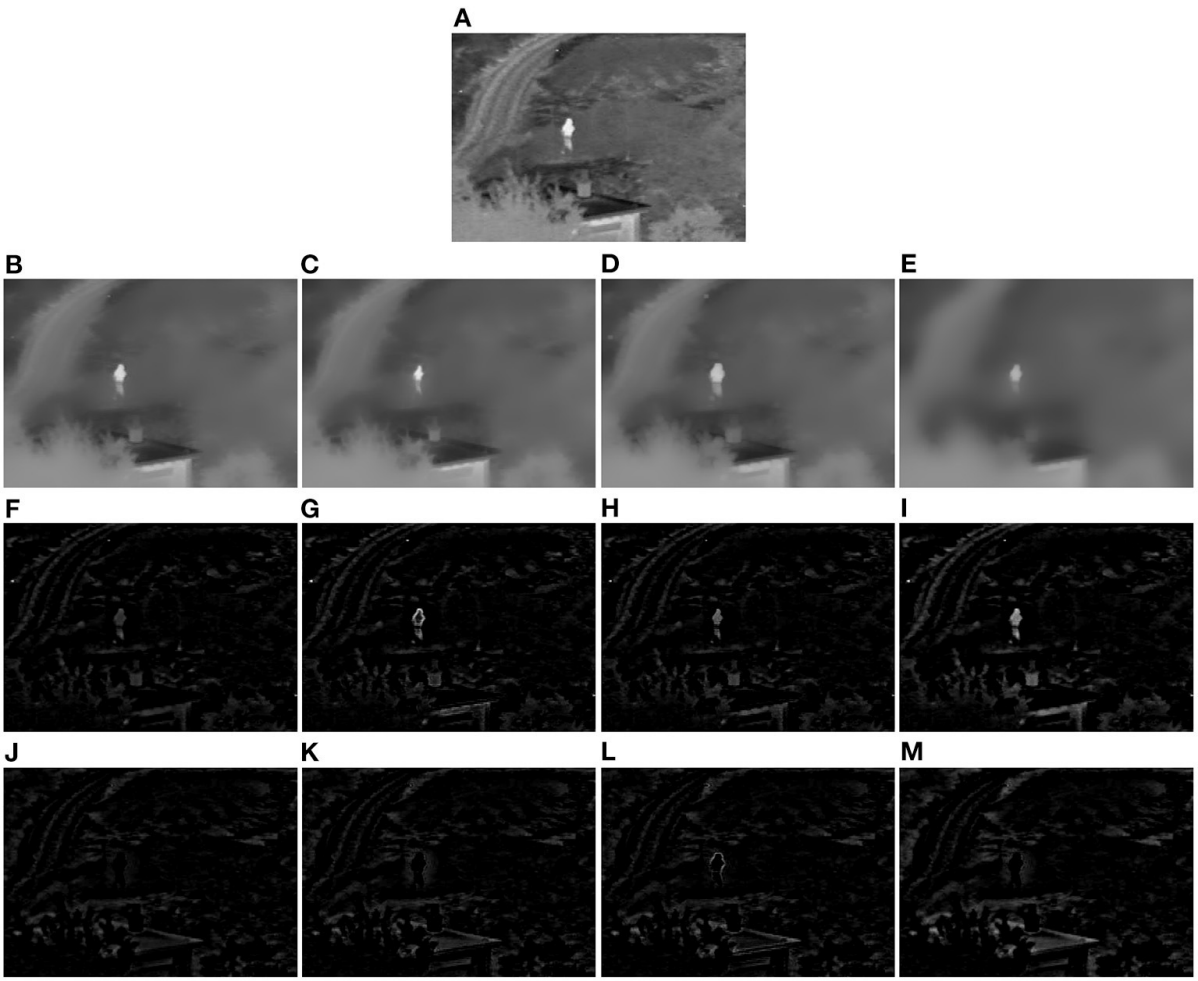
Demonstration example of the local extreme map guided image filter (Toet, 2017). **(A)** Original infrared image. **(B)** Image smoothed by the image filter guided by the input image itself. **(C)** Image smoothed by single local minimum map guided image filter. **(D)** Image smoothed by single local maximum map guided image filter. **(E)** Image smoothed by our local extreme map guided image filter. **(F–I)** Bright feature maps extracted from the difference images of **(B–E)** and **(A)**, respectively. **(J–M)** Dark feature maps extracted from the difference images of **(B–E)** and **(A)**, respectively (Note that the dark feature maps in this figure have been illustrated as their absolute feature maps in order to properly visualize the dark features).

As is known, there exist both bright features and dark features in an image, such as the bright person and the dark roof in Figure 2A. Through sequentially smoothing the input image guided by the local minimum map and local maximum map, respectively, both the salient bright and dark features will be removed from the input image and a well-smoothed image will be obtained. Then, the salient features of the input image can be obtained by subtracting the filtered image *I*_*f*_ from the input image *I* according to Equation (4), and the positive part of (*I*−*I*_*f*_) corresponds to the bright features, and the negative part corresponds to the dark features.


(4) $$\{\begin{array}{c} F_{b}=\text{max }\left(I-I_{f},0\right)\\ F_{d}=\text{min }\left(I-I_{f},0\right) \end{array},$$

where *F*_*b*_ and *F*_*d*_ denote the bright feature map and dark feature map of *I*, respectively.

A demonstration example of the proposed local extreme map guided image filter performed on an infrared image is illustrated in Figure 2. In this figure, we have compared the smoothed images (see Figures 2B–E), respectively, by the original guided image filter, single local minimum map guided filter, single local maximum map guided filter, and our complete extreme map guided filter, and also compared the feature maps extracted from their difference images with respect to the original infrared image in Figure 2A. It can be seen from Figures 2B–E that the smoothed image by our extreme map guided filter has suppressed more salient features (textural details) compared to those of the original guided filter, single local minimum map guided filter, and single local maximum map guided filter. Accordingly, the salient features (see Figures 2I,M) extracted by our extreme map guided filter are far more than those extracted by the original guided filter, single local minimum map guided filter, and single local maximum map guided filter. Moreover, intensities of our extracted feature maps are much higher than those of feature maps extracted by the other three filters. Overall, the results in this figure suggest that our constructed local extreme map guided filter is able to extract the input image's bright and dark features well and significantly outperforms the original guided filter, single local minimum map guided filter, and single local maximum map guided filter.

Naturally, the local extreme map guided image filter can be extended to multiple scales by iteratively applying the image filter guided with local minimum map and that guided with local maximum map on the input image *I* according to Equation (5).


(5) $$\begin{array}{l} I_{f}^{i}=leguidedfilter\;\left(I_{f}^{\left(i-1\right)},se_{i},r_{i}\right), \end{array}$$

where *i* denotes the current scale of the guided filter, and *i* is increased from 1 to *n* one by one. $I_{f}^{i}$ denotes the *i*th-scale filtered image and especially $I_{f}^{0}$ is the original input image *I*. *se*_*i*_ and *r*_*i*_ denote the structuring element and size of the local window at the *i*th scale, respectively.

Accordingly, different scales of bright and dark features can be simultaneously extracted from the difference image of every two continuously filtered images according to Equation (6).


(6) $$\{\begin{array}{c} F_{b,i}=\max\;\left(I_{f}^{i-1}-I_{f}^{i},0\right)\\ F_{d,i}=\min\;\left(I_{f}^{i-1}-I_{f}^{i},0\right) \end{array}.$$

Finally, the last scale of filtered image is taken as the base image of *I*, as expressed in Equation (7).


(7) $$\begin{array}{l} I_{base}=I_{f}^{i}. \end{array}$$

### 2.2. Local extreme map guided image fusion

In this study, we aim to fuse two multi-modal brain images (denoted by *I*^1^ and *I*^2^). According to the feature extraction method introduced in the previous subsection, we can well extract the multi-scale bright feature maps (denoted by $F_{b,i}^{j}$) and dark feature maps (denoted by $F_{d,i}^{j}$) of each input image *I*^*j*^, and simultaneously obtain their base images (denoted by $I_{base}^{j}$). *j* denotes index of the input image, and is ranged from 1 to 2. Then, the detailed procedures for fusing two multi-modal brain images are introduced as follows.

As the high-frequency features of high intensities are usually corresponding to the salient sharp features in the image, thus we fuse each scale of bright feature maps of the two multi-modal brain images by selecting their elementwise-maximum values and fuse each scale of dark feature maps of the two multi-modal images as their elementwise-minimum values as:


(8) $$\{\begin{array}{c} F_{b,i}^{fuse}=\text{max }\left(F_{b,i}^{1},F_{b,i}^{2}\right)\\ F_{d,i}^{fuse}=\text{min }\left(F_{d,i}^{1},F_{d,i}^{2}\right) \end{array},$$

Like other feature extractors, the proposed local extreme map guided image filter cannot extract the entire bright and dark features from the source images either, thus we have enhanced the fused bright and dark features by multiplying each scale of fused bright feature map and dark feature map by an information-amount related weight. Further, the enhanced bright feature maps and dark feature maps are integrated, respectively. The above two procedures can be mathematically expressed as:


(9) $$\{\begin{array}{l} F_{b}^{fuse}=\sum_{i=1}^{n}w_{b,i}\cdot F_{b,i}^{fuse}\\ F_{d}^{fuse}=\sum_{i=1}^{n}w_{d,i}\cdot F_{d,i}^{fuse} \end{array},$$

where *w*_*b, i*_ denotes the weight of the *i*th scale of bright feature map and *w*_*d, i*_ denotes the weight of the *i*th scale of dark feature map. Generally, the feature map with more information should be assigned to a large weight, thus *w*_*b, i*_ and *w*_*d, i*_ are set according to the entropy of $F_{b,i}^{fuse}$ and $F_{d,i}^{fuse}$, respectively, as:


(10) $$\{\begin{array}{c} w_{b,i}=\frac{e_{b,i}}{\underset{j}{\min}\left(e_{b,j}\right)}\\ w_{d,i}=\frac{e_{d,i}}{\underset{j}{\min}\left(e_{d,j}\right)} \end{array},$$

where *e*_*b, i*_ denotes the entropy of $F_{b,i}^{fuse}$ and *e*_*d, i*_ denotes the entropy of $\left(-F_{d,i}^{fuse}\right)$. In this way, the minimum weight, i.e., weight of feature map with the lowest entropy, will be 1, and weights of other scales of feature maps will all be higher than 1. Accordingly, most scales of bright and dark feature maps will be enhanced to some degree according to their information amount.

As for the low-frequency base images, we directly fuse them by computing their elementwise-maximum values according to Equation (11). In this manner, most basic information of the multi-modal medical images will preserved into the final fusion image.


(11) $$I_{base}^{fuse}=\text{max }\left(I_{base}^{1},I_{base}^{2}\right).$$

Finally, the fusion image can be generated by combining the fused bright feature map, dark feature map, and base image together as expressed in Equation (12). In this way, our fused image can not only preserve as much as basic information of the multi-modal source images, but also well enhance the salient sharp features of the multi-modal source images.


(12) $$\begin{array}{l} I^{fuse}=F_{b}^{fuse}+F_{d}^{fuse}+I_{base}^{fuse}. \end{array}$$

### 2.3. Parameter settings

In our method, there are mainly three parameters, including the scale number *n*, the size of the local window *r*_*i*_ in the guided image filter, and the radius of the structuring element *k*_*i*_ in the morphological erosion and dilation operations. In order to balance the time cost and fusion effect of the multi-modal brain images, *n* is set to five in this study, i.e., *n* = 5. As for *r*_*i*_ and *k*_*i*_, we keep them same with each other, i.e., *k*_*i*_ = *r*_*i*_, in in each iteration *i* of local extreme map guided image filtering. Moreover, in order to effectively extract the salient image features, we set *r*_*i*_ = 2×*i*+1 where *i* is gradually increased from 1 to *n* in this study. The extensive experimental results verify the above settings are effective for fusing the multi-modal brain images.

## 3. Experimental results and discussions

In order to verify the effectiveness of the proposed image fusion method, we have compared it with eight representative image fusion methods on three commonly used multi-modal brain image datasets (Xu and Ma, 2021). The detailed experimental settings, implementation details, results, and discussions are introduced in the following five subsections.

### 3.1. Experimental settings

**Figure 3:**
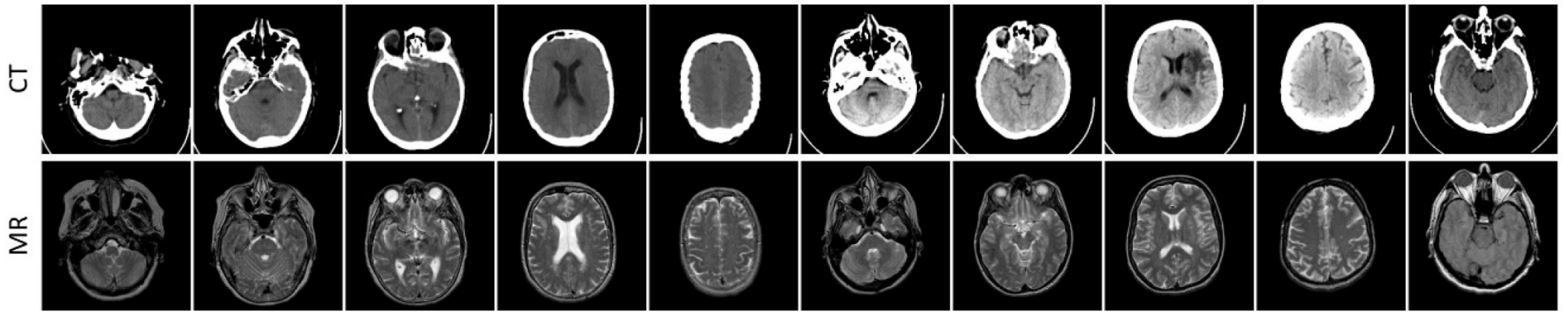
Ten pairs of images in the CT-MR image dataset.

**Figure 4:**
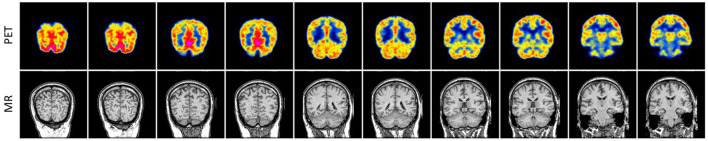
Ten pairs of images in the PET-MR image dataset.

**Figure 5:**
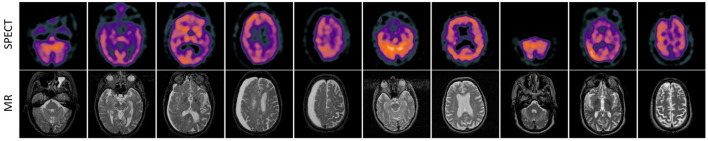
Ten pairs of images in the SPECT-MR image dataset.

At first, we take 30 pairs of commonly used multi-modal brain images from http://www.med.harvard.edu/aanlib as our testing sets, including 10 pairs of CT and MR brain images, 10 pairs of PET and MR images, and 10 pairs of SPECT and MR images. The three used datasets have been shown in Figures 3–5, respectively. In particular, the spatial resolution of the images in the three datasets are all 256 × 256.

Second, we have compared our method with eight SOTA image fusion methods, including the discrete wavelet transform based method (DWT) (Li et al., 1995), the guided-filter based method (GFF) (Li et al., 2013), the Laplacian pyramid and sparse representation base method (LPSR) (Liu et al., 2015), the unified image fusion network (U2Fusion) (Xu et al., 2020a), the GAN based method (DDcGAN) (Ma et al., 2020), the general CNN based image fusion network (IFCNN) (Zhang et al., 2020), the enhanced medical image fusion network (EMFusion) (Xu and Ma, 2021), and the disentangled representation based brain image fusion network (DRBIF) (Wang et al., 2022). Moreover, in order to verify the efficacy of the guidance of local extreme maps, we have also added our method without the guidance of local extreme maps (denoted by LEGFF_0_) for comparison.

At last, qualitative evaluation heavily depends on the subjective observation which is inaccurate and laborious, thus 11 commonly-used quantitative metrics are further used to objectively compare the 10 methods' performance. The 11 quantitative metrics are spatial frequency (SF) (Li and Yang, 2008), average absolute gradient (AbG), perceptual saliency (PS) (Zhou et al., 2016), standard deviation (STD), entropy (E), Chen-Blum Metric (QCB) (Chen and Blum, 2009), visual information fidelity (VIFF) (Han et al., 2013), edge preservation metric (Qabf) (Xydeas and Petrovic, 2000), gradient similarity metric (QGS) (Liu et al., 2011), weighted structural similarity metric (WSSIM) (Piella and Heijmans, 2003), and multi-scale structural similarity (NSSIM) (Ma et al., 2015). Among these metrics, SF, AG, PS, and STD quantify the amount of details reserved in the fusion image, E measures the intensity distribution of the fusion image, QCB measures the amount of the preserved contrast information of the fusion image compared to the source images, VIFF measures the information fidelity of the fusion image with respect to the source images, Qabf measures the amount of the preserved edge information of the fusion image compared to the source images, QGS measures the gradient similarity of the fusion image and the corresponding source images, and WSSIM and NSSIM both measure the structural information of the fusion image preserved from the source images. Overall, the 11 selected metrics can quantitatively evaluate the fusion images of different image fusion methods from various aspects, and the larger values of all the 11 metrics indicate the better performance of the corresponding image fusion method.

### 3.2. Implementation details

Among the 10 comparison methods, IFCNN and DRBIF can be directly used to fuse color images, and the other eight fusion methods can only fuse gray-scale images directly. Thus, DWT, GFF, LPSR, U2Fusion, DDcGAN, EMFusion, LEGFF_0_, and our method can be directly applied to fuse the pair of gray-scale CT and MR images in the CT-MR image dataset. As for fusing images in the PET-MR and SPECT-MR image datasets, the color image (PET or SPECT image) is first transformed from the RGB color space to the YCbCr color space. Then, these eight methods fuse the Y channel of the color image and the gray-scale MR image together. Finally, the fused color image is generated by concatenating the fused gray-scale image and Cb and Cr channels of the original color image, and transforming the fused image in the YCbCr color space back to the RGB color space. Moreover, most quantitative metrics are designed to quantify the quality of gray-scale fusion images. Thus, during computing the quantitative metric values on the PET-MR and SPECT-MR image datasets, we covert the color source image and the corresponding color fusion image to the YCbCr color space and take their Y channels to compute the metric value of this color fusion image. Finally, code of our proposed method will be released on https://github.com/uzeful/LEGFF.

### 3.3. Qualitative evaluation results

**Figure 6:**
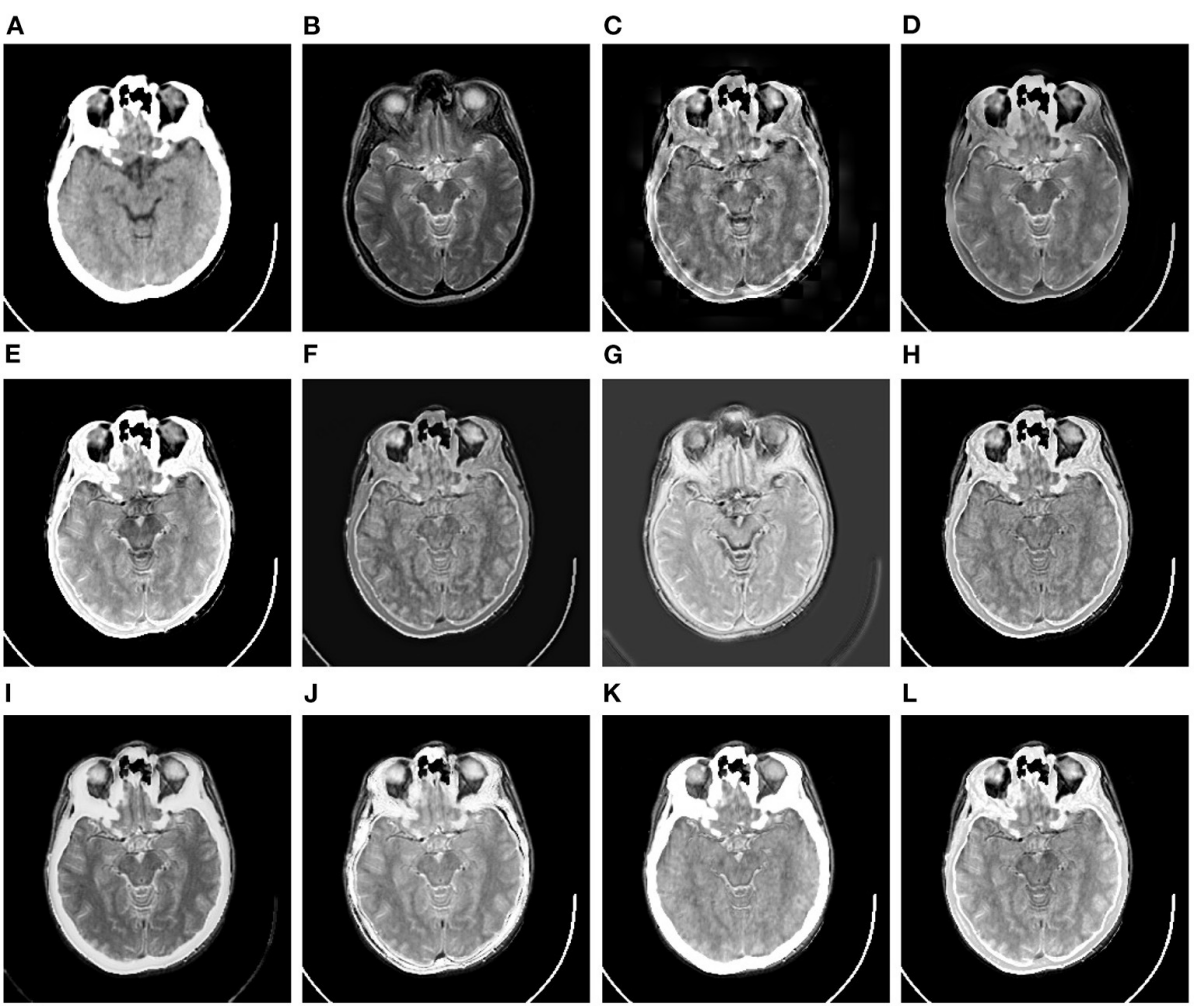
Comparison example on the CT-MR image dataset. **(A,B)** are the original CT image and MR image, respectively. **(C–L)** are the fusion results of DWT, GFF, LPSR, U2Fusion, DDcGAN, IFCNN, EMFusion, DRBIF, LEGFF_0_, and our method, respectively.

**Figure 7:**
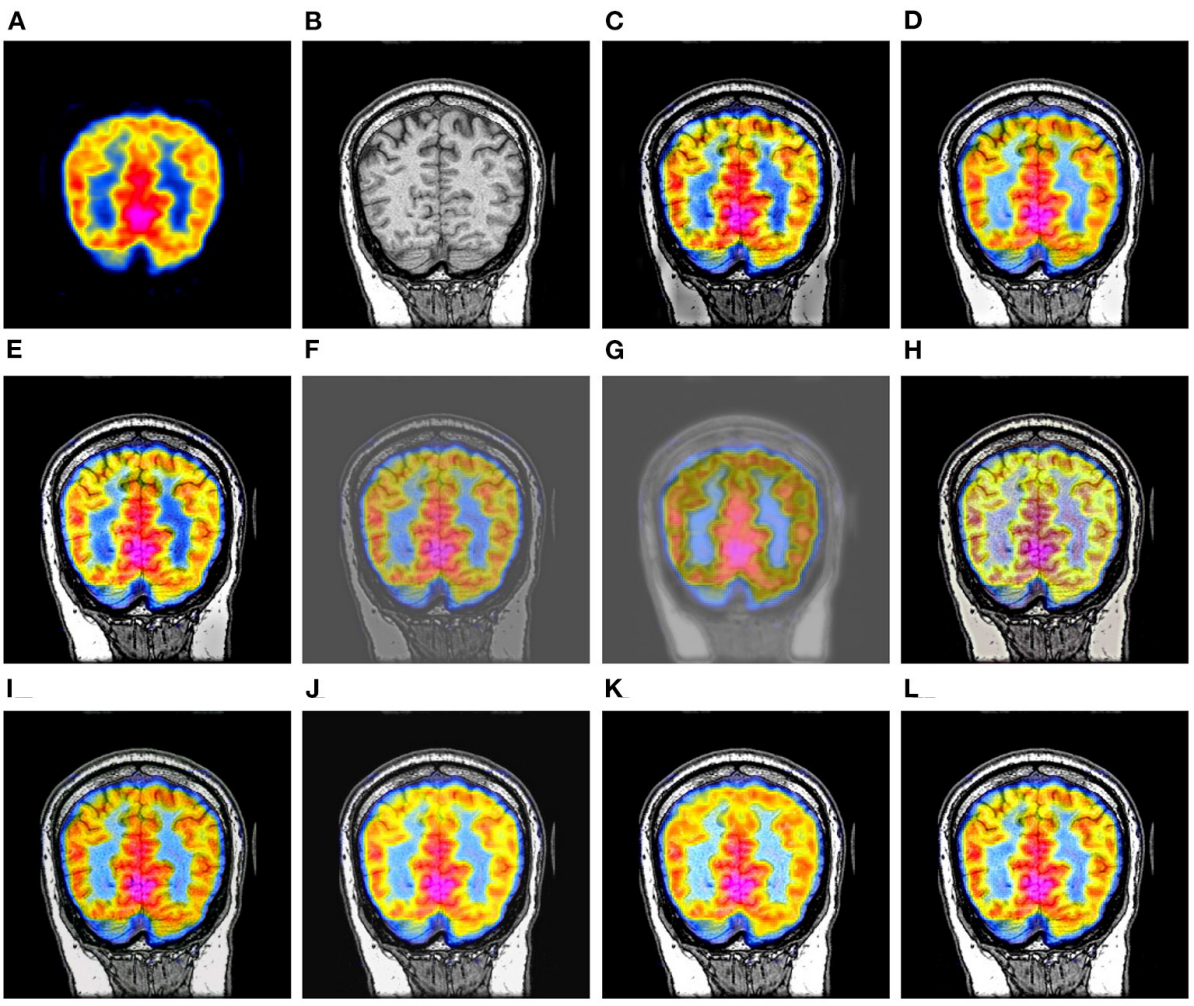
Comparison example on the PET-MR image dataset. **(A,B)** are the original PET image and MR image, respectively. **(C–L)** are the fusion results of DWT, GFF, LPSR, U2Fusion, DDcGAN, IFCNN, EMFusion, DRBIF, LEGFF_0_, and our method, respectively.

**Figure 8:**
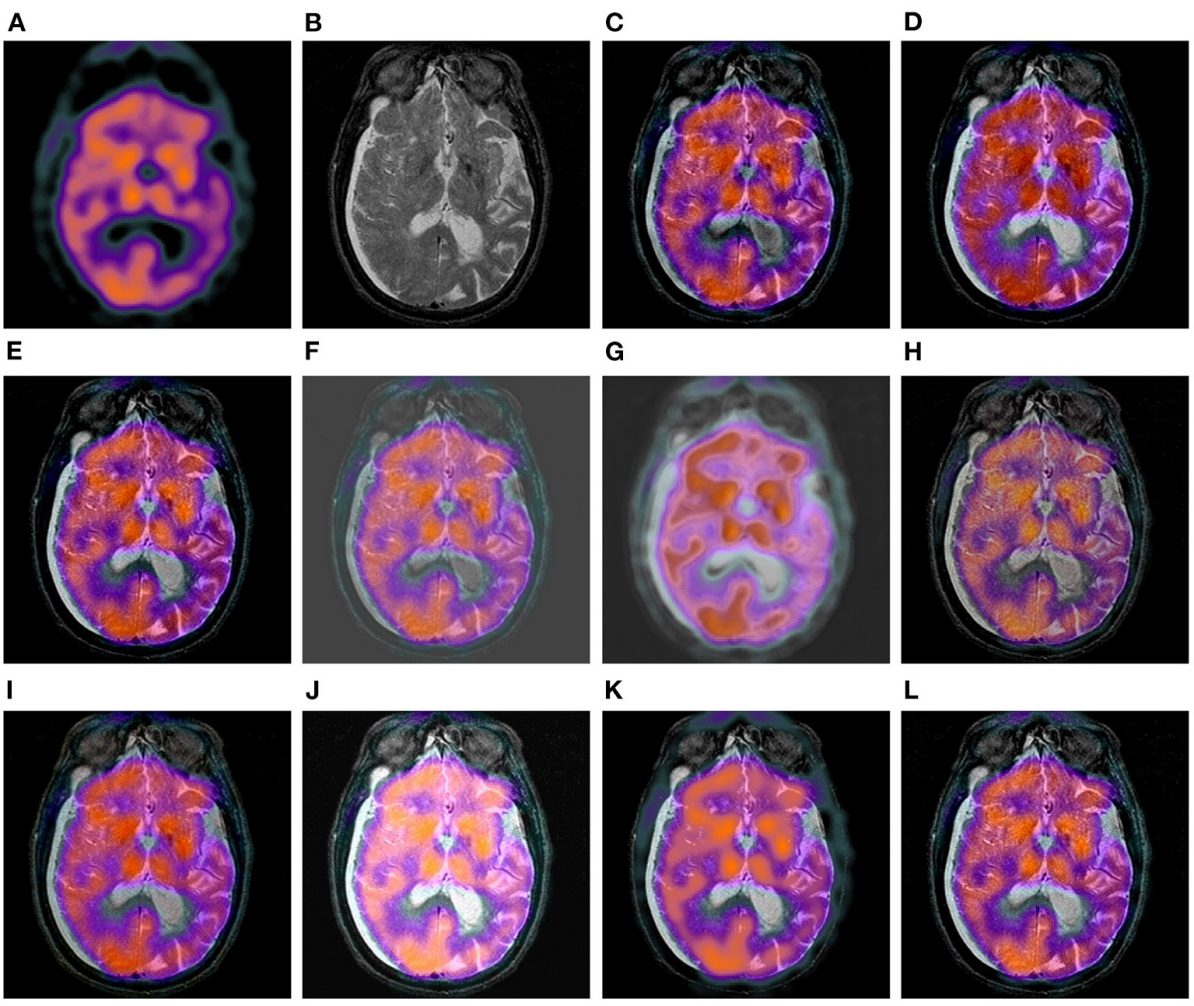
Comparison example on the SPECT-MR image dataset. **(A,B)** are the original SPECT image and MR image, respectively. **(C–L)** are the fusion results of DWT, GFF, LPSR, U2Fusion, DDcGAN, IFCNN, EMFusion, DRBIF, LEGFF_0_, and our method, respectively.

In this subsection, the 10 image fusion methods are evaluated by the qualitative method, i.e., comparing their fusion results through visual observation. Specifically, we have shown three comparison examples of the 10 image fusion methods in Figures 6–**8**, respectively.

Figure 6 shows a set of fusion results of the 10 image fusion methods on the CT-MR image dataset. It can be seen from Figure 6C that the fusion image of DWT demonstrates severe blocking effect around the head. Figures 6D,F reflect that the fusion images of GFF and U2Fusion are of relatively low contrast. Figure 6G shows that the background of the DDcGAN's fusion image becomes gray and leads to low-contrast effect. It can be seen from Figures 6I,K that EMFusion and LEGFF_0_ fail to integrate the textures of soft tissues in the skull region of the original MR image into their fusion images. Figure 6J shows that DRBIF fails to integrate several parts of skull region of the original CT image into its fusion image. Finally, the fusion images of LPSR, IFCNN, and our method in Figures 6E,H,J achieve the best visual effect among all the fusion images, i.e., having better contrast and integrating the salient textures of the original MR image and CT image into their fusion images.

Figure 7 shows a set of fusion results of the 10 image fusion methods on the PET-MR image dataset. It can be seen from Figure 7C that the intensities of the bottom part of DWT's fusion image are significantly lower than that of the original MR image in Figure 7B. Figure 7E shows that the intensities of the bottom right of LPSR's fusion image are slightly lower than that of the original MR image in Figure 7B. The fusion results of U2Fusion and DDcGAN in Figures 7F,G have much lower contrast than those of other methods. Figure 7H shows that the color style of IFCNN's fusion image is significantly changed as compared to that of the original PET image in Figure 7A. Figures 7J,K show that DRBIF and LEGFF_0_ fail to integrate some dark features of the MR image into their fusion images. Overall, the fusion images of GFF, EMFusion, and our method in Figures 7D,I,L integrate most salient features of the original PET and MR images into their fusion images, but contrast of EMFusion's fusion image is a little lower than that of GFF's fusion image and ours.

Figure 8 shows a set of fusion results of the 10 image fusion methods on the SPECT-MR image dataset. We can see from Figure 8C that the fusion image of DWT loses a few textures around the center regions of the two eyes. It can be seen from Figures 8D,E that GFF and LPSR only integrate a few details of the bottom skull region of the original MR image into their fusion images. The fusion image of U2Fusion and DDcGAN in Figures 8F,G still have the defect of lower contrast and gray background. The fusion image of IFCNN in Figure 8H is of low contrast compared to the original SPECT and MR images in Figures 8A,B. Figure 8J shows that the color style of DRBIF's fusion image is significantly different from that of the original SPECT image in Figure 8A and DRBIF fails to integrate a few bright features of the original MR image into its fusion image due to its relatively high intensity. Figure 8K shows that LEGFF_0_ fails to integrate many bright features of the original MR image into its fusion image. Overall, the fusion images of DWT, EMFusion, and our method in Figures 8C,I,L exhibit the best visual effects among all the fusion images, but the salient features integrated in our fusion image are more complete than those integrated in the fusion images of DWT and EMFusion.

The three comparison examples could verify that the proposed method can effectively fuse the salient bright and dark features of the multi-modal brain images into a comprehensive fusion image, and outperforms the eight SOTA image fusion methods according to the visual comparison results. Moreover, through visually comparing the fusion results of LEGFF_0_ and our method, it could be verified that the incorporation of the local extreme map guidance is critical for improving the feature extraction ability and feature fusion ability of the guided image filter.

### 3.4. Quantitative evaluation results

The quantitative metric values of the eight image fusion methods are first calculated according to their fusion results on each dataset, then the average metric values of the eight methods on each dataset are listed in Tables 1–**3**, respectively. In each table, the values in the **bold**, underline, and *italic* fonts indicate the best, second-best and third-best results, respectively.

**Table 1:** Quantitative evaluation results on the CT-MRI dataset.

**Metrics**	**Methods**									
	**DWT**	**GFF**	**LPSR**	**U2Fusion**	**DDcGAN**	**IFCNN**	**EMFusion**	**DRBIF**	**LEGFF0**	**Ours**
SF	*33.6948*	28.4859	**35.7214**	22.6498	21.2063	34.0565	21.8423	29.4565	31.3377	32.8535
AbG	16.6486	12.8718	14.7253	12.7615	13.8349	**16.7553**	11.7355	14.5474	12.8055	*15.1338*
STD	69.4181	62.5530	86.7440	55.0136	63.6475	76.6713	76.1188	83.2861	**89.8708**	*84.8222*
QPS	41.6377	35.8390	**47.8699**	30.9477	29.9360	43.2325	35.6807	42.0657	*44.8173*	44.9017
E	**5.0467**	4.3135	3.7990	4.6787	4.9987	4.2717	*4.5214*	4.3620	3.1752	4.2705
QCB	0.5436	0.6598	**0.7074**	0.2906	0.1683	0.6843	*0.6699*	0.6562	0.6603	0.6342
VIFF	0.3539	0.2733	0.4280	0.3185	0.2002	0.4256	0.3992	*0.4318*	0.4539	**0.4800**
Qabf	0.5538	0.7319	0.7394	0.6503	0.5934	0.7598	0.7247	*0.7461*	**0.7821**	0.7170
QGS	0.8796	0.8521	*0.9000*	0.8088	0.7776	**0.9135**	0.8053	0.8580	0.8766	0.9054
WSSIM	0.7113	0.8245	0.8178	0.3525	0.1931	**0.8456**	0.8088	*0.8266*	0.8139	0.8415
MSSIM	0.8731	0.8460	0.9395	0.8736	0.6584	*0.9384*	0.8819	0.9095	0.8926	**0.9505**

It can be seen from Table 1 that the proposed method has achieved the best performance on two metrics (i.e., VIFF and NSSIM), obtained second-best performance on three metrics (i.e., PS, QGS, and WSSIM), ranked the third place on the STD metric on the CT-MR image dataset. To be specific, the largest VIFF and NSSIM values and second-largest QGS and WSSIM values of our method suggest that our fusion images have preserved relatively more edge and structural information from the original CT and MR images than the fusion images of other methods. The second-largest PS value and the third-largest STD value of our method indicate that the fusion images of our method have slightly more textural details than those generated by the other eight comparison methods. Since in our method the base images of the source images are fused as their elementwise-maximum values, thus intensity distribution of our fusion images might be not that uniform along the gray-scale space leading to relatively lower E and QCB values. Besides our method, LPSR has achieved the best performance on three metrics (i.e., SF, PS, and QCB) and the second performance on two metrics (i.e., STD and MSSIM) and IFCNN has achieved the best performance on three metrics (i.e., AbG, QGS, and WSSIM) and the second performance on three metrics (i.e., SF, QCB, and Qabf). Overall, consistent to the qualitative comparison results, the quantitative evaluation results in Table 1 shows LPSR, IFCNN, and our method perform slightly better than the other seven methods on fusing the CT and MR images.

Table 2 shows that our method has achieved the best performance on eight metrics (i.e., SF, AbG, PS, QCB, VIFF, Qabf, QGS, and WSSIM) and the second-best performance on two metrics (i.e., STD and MSSIM). As addressed previously, the E metric value of our method is relatively lower than those of other methods, due to our usage of the elementwise-maximum strategy for fusing the base images. Overall, the quantitative evaluation results on the PET-MR image dataset suggest our method significantly outperforms the other nine methods by a large margin in particular on fusing the PET and MR images. This conclusion is also consistent to the visual comparison results from Figure 7.

**Table 2:** Quantitative evaluation results on the PET-MRI dataset.

**Metrics**	**Methods**									
	**DWT**	**GFF**	**LPSR**	**U2Fusion**	**DDcGAN**	**IFCNN**	**EMFusion**	**DRBIF**	**LEGFF0**	**Ours**
SF	33.5860	35.0668	34.5411	10.0922	6.2573	33.4545	29.6977	28.2939	*34.7476*	**38.0866**
AbG	22.2784	*22.6448*	22.4732	7.2796	4.5950	22.9097	20.2215	18.6718	21.5265	**25.0111**
STD	68.8455	*74.7923*	73.3129	27.8267	24.9354	72.8165	68.6928	72.6768	**80.3887**	79.2849
QPS	36.7423	*40.6313*	39.2959	13.9280	10.4126	38.0456	34.6725	35.4353	41.2365	**42.5936**
E	4.8620	4.9216	4.8230	4.6586	4.9882	*5.1515*	5.3884	**5.4553**	4.5049	4.8948
QCB	0.5747	0.5968	0.6075	0.3435	0.2272	*0.5997*	0.5989	0.3475	0.5683	**0.6145**
VIFF	0.4784	0.4248	*0.4926*	0.1779	0.0446	0.4969	0.4018	0.4702	0.4403	**0.5175**
Qabf	0.6443	*0.7340*	0.7070	0.4017	0.3277	0.7118	0.7171	0.6761	0.7346	**0.7392**
QGS	0.8905	0.9256	0.9123	0.7595	0.7164	*0.9142*	0.9015	0.8954	0.9128	**0.9344**
WSSIM	0.6754	0.7170	0.7027	0.3623	0.1611	0.7098	0.7052	0.6569	0.6708	**0.7201**
MSSIM	0.9238	0.9003	**0.9463**	0.6364	0.3814	*0.9387*	0.8983	0.9216	0.8817	0.9436

Finally, it can be seen from Table 3 that our method has ranked the first place on six metrics (i.e., SF, AbG, QCB, Qabf, WSSIM, and MSSIM), ranked the second place on the QPS, VIFF, and QGS metrics, and ranked the third place on the STD metric. Besides, DRBIF have obtained the best performance on five metrics (i.e., STD, QPS, E, VIFF, and QGS) and the second place on three metrics (i.e., SF, AbG, and MSSIM). These results suggest the fusion images of our method and DRBIF have more textural details and persevered more structural information from the original SPECT and MR images compared to those of the other eight methods. Moreover, the quantitative results in Tables 1–3 indicate that our method with the local extreme map guidance significantly outperforms that without the local extreme map guidance. Thus, the incorporation of the local extreme map guidance is effective for fusing the multi-modal medical images.

**Table 3:** Quantitative evaluation results on the SPECT-MRI dataset.

**Metrics**	**Methods**									
	**DWT**	**GFF**	**LPSR**	**U2Fusion**	**DDcGAN**	**IFCNN**	**EMFusion**	**DRBIF**	**LEGFF0**	**Ours**
SF	16.4700	16.4841	*16.6859*	8.0194	6.4764	15.6082	13.2987	17.1988	14.7571	**18.5201**
AbG	11.4319	11.0090	11.3386	5.7527	4.9230	*11.7558*	9.6794	12.7670	9.0886	**13.4389**
STD	40.8780	46.4581	47.2084	24.2069	39.5366	42.6516	42.9472	**64.6827**	49.2280	*48.9651*
QPS	21.4775	23.2906	*23.4398*	12.5464	12.7030	20.3133	18.7972	**26.1537**	21.7281	24.3273
E	4.4663	4.2638	4.4474	4.5896	5.6676	5.1628	*5.2005*	**5.7996**	4.3966	4.9760
QCB	0.5633	0.5926	*0.5858*	0.3456	0.2249	0.5838	0.5807	0.3706	0.5380	**0.6000**
VIFF	0.4749	0.4230	*0.4873*	0.2327	0.2141	0.4814	0.4411	**0.7241**	0.4531	0.5646
Qabf	0.5656	0.6269	0.6137	0.3996	0.2824	0.6860	0.6461	*0.6501*	0.5976	**0.6866**
QGS	0.9194	0.9332	*0.9368*	0.8646	0.8368	0.9353	0.9184	**0.9461**	0.8984	0.9411
WSSIM	0.6371	0.6509	0.6546	0.4302	0.2113	*0.6512*	0.6425	0.5305	0.5936	**0.6548**
MSSIM	0.9299	0.8973	0.9411	0.7797	0.5437	*0.9475*	0.9372	0.9496	0.8949	**0.9556**

**Figure 9:**
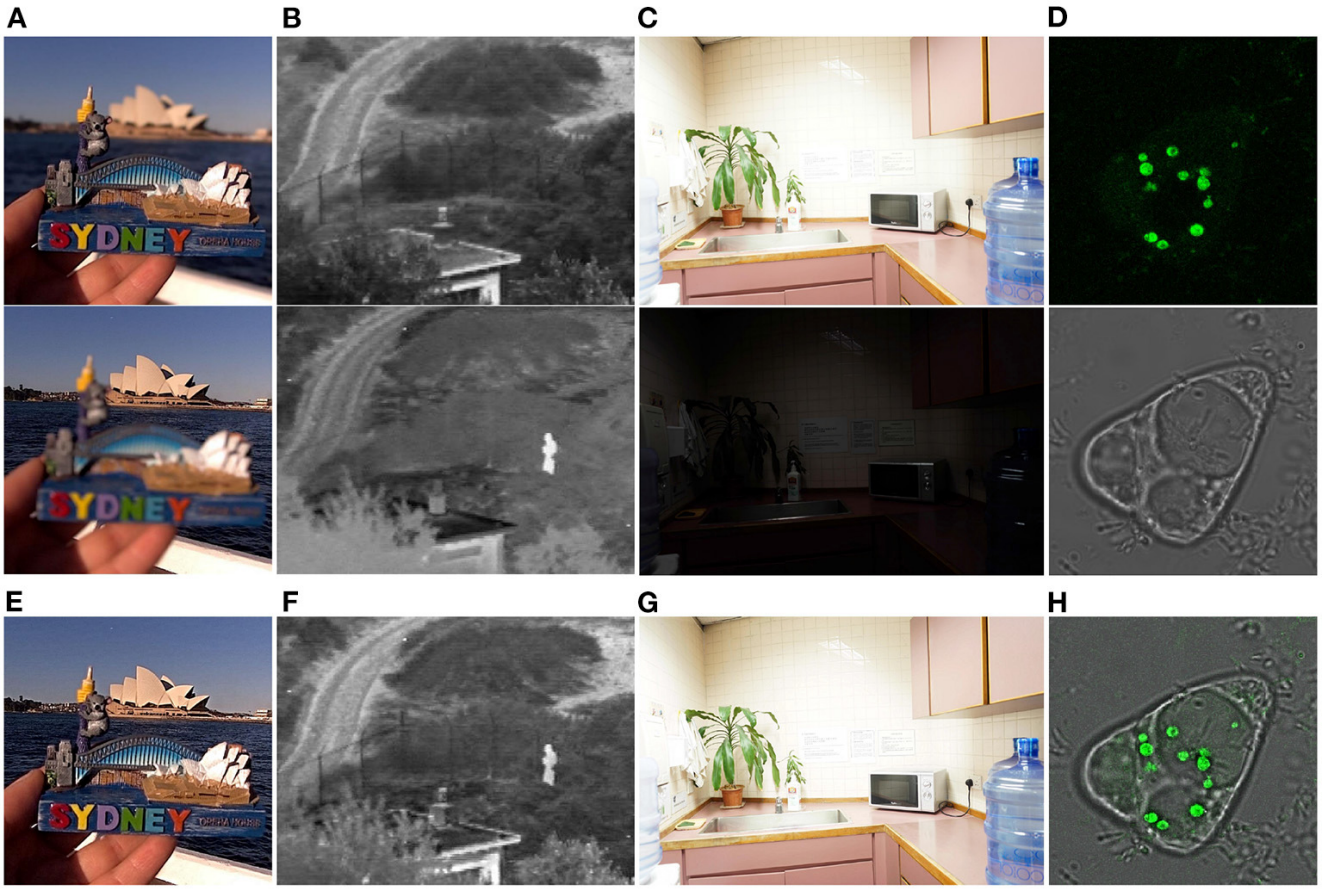
Our fusion results on other modalities of images. **(A)** Shows a pair of multi-focus images (Nejati et al., 2015). **(B)** Shows a pair of visual and infrared images (Toet, 2017). **(C)** Shows a pair of over- and under-exposed images (Xu et al., 2020b). **(D)** Shows a pair of green-fluorescent and phase-contrast protein images (Tang et al., 2021). **(E–H)** are the fusion results of **(A–D)**, respectively.

Besides, in order to test the efficiency of our proposed method, we have compared the average time cost of each method on the SPECT-MRI image dataset. All methods were evaluated on the same computation platform with Intel Core i7-11700K CPU and NVIDIA GeForce RTX 3090 GPU. The evaluation results have been listed in Table 4. It can be seen from Table 4 that LPSR and DWT run much faster than the other methods. As for our method, it costs about 0.1230 s to fuse a pair of multi-modal brain images, and it is slightly faster than three deep learning based methods including U2Fusion, DDcGAN, and EMFusion. Therefore, in term of time cost evaluation, the proposed method is relatively time-efficient as compared to the other nine comparison methods. Moreover, in order to verify the generalization ability of our method, we have apply it to fuse other modalities of images, including the multi-focus images, infrared and visual images, multi-exposure images, and green-fluorescent and phase-contrast protein images. Figure 9 shows that our method can well integrate the salient features of each pair of source images into the corresponding fusion images. Thus, the good fusion results in Figure 9 can verify the good generalization ability of our method for fusing other modalities of images. Overall, both qualitative and quantitative evaluation results indicate that our method performs comparably to or even better than eight SOTA image fusion methods and owns good generalization ability.

**Table 4:** Time cost comparison.

**Methods**	**DWT**	**GFF**	**LPSR**	**U2Fusion**	**DDcGAN**	**IFCNN**	**EMFusion**	**DRBIF**	**LEGFF0**	**Ours**
Time costs	0.0077	0.1035	**0.0021**	0.3019	0.7935	*0.0391*	0.1323	0.0997	0.0401	0.1230

### 3.5. Limitations and future prospects

Even though the experimental results validate the advantages of our image fusion method, there still exist several limitations in our method. At first, our local extreme map guided image filter is constructed on the basis of the guided image filter, thus the feature extraction ability of our filter will be inevitably impacted by that of the original guided image filter. Second, compared to LEGFF_0_ (which uses the original guided filter solely for feature extraction and image fusion), the time cost of our image fusion method increases by a large margin due to iterative calculation of local extreme maps. In future, with the development of guided image filter, performance of our image fusion method can be further boosted by incorporating more advanced guided image filter. Moreover, integrating the local extreme map guidance and the deep-learning frameworks is another way to simultaneously improve the performance and efficiency of the local extreme map guided image fusion methods. Finally, the proposed image fusion method does not contain the image denoising and registration procedures, thus before applying our method in the clinical scenarios the pair of multi-modal source images should be denoised and aligned first.

## 4. Conclusion

In this study, we propose an effective multi-modal brain image fusion method based on a local extreme map guided image filter. The local extreme map guided image filter can well smooth the image, thus it can further be used to extract the salient bright and dark features of the image. By iteratively applying this local extreme map guided image filter, our method is able to extract multiple scales of bright and dark features from the multi-modal brain images, and integrate these salient features into one informative fusion image. Extensive experimental results suggest that the proposed method outperforms eight SOTA image fusion methods from both qualitative and quantitative aspects and it demonstrates very good generalization ability to fuse other modalities of images. Therefore, the proposed method exhibits great possibility to apply in the real clinical scenarios.

## Data availability statement

Publicly available datasets were analyzed in this study. This data can be found here: http://www.med.harvard.edu/aanlib. Code and used images are available at https://github.com/uzeful/LEGFF.

## Author contributions

YZ and QZ designed the study. YZ, WX, SZ, and JS performed data analysis. YZ wrote the manuscript. QZ, LZ, XB, and RW revised the manuscript. All authors contributed to the article and approved the final submitted version.

## Funding

This study was supported in part by the National Key Research and Development Program of China under Grant No. 2019YFB1311301, in part by the National Natural Science Foundation of China under Grant Nos. 62171017, 61871248, 62132002, 62173005, and 61976017, in part by the Beijing Natural Science Foundation under Grant No. 4202056, and in part by China Postdoctoral Science Foundation under Grant No. 2021M690297.

## Conflict of interest

The authors declare that the research was conducted in the absence of any commercial or financial relationships that could be construed as a potential conflict of interest.

## Publisher's note

All claims expressed in this article are solely those of the authors and do not necessarily represent those of their affiliated organizations, or those of the publisher, the editors and the reviewers. Any product that may be evaluated in this article, or claim that may be made by its manufacturer, is not guaranteed or endorsed by the publisher.
